# Myeloid leukemia factor 1: A “double-edged sword” in health and disease

**DOI:** 10.3389/fonc.2023.1124978

**Published:** 2023-02-06

**Authors:** Zixuan Li, Yuanyuan Yang, Kun Wu, Yuntao Li, Mingxia Shi

**Affiliations:** ^1^ Department of Hematology, the First Affiliated Hospital of Kunming Medical University, Kunming, China; ^2^ Hematology Research Center of Yunnan Province, Kunming, China; ^3^ Department of Clinical Laboratory, First Affiliated Hospital of Kunming Medical University, Kunming, China

**Keywords:** myeloid leukemia factor 1, nucleo-cytoplasmic shuttling protein, cell cycle regulation, immune function, cell development and differentiation, malignancy development

## Abstract

The occurrence and development of malignancies are closely related to abnormal cell cycle regulation. Myeloid leukemia factor 1 (MLF1) is a small nucleocytoplasmic shuttling protein associated with cell cycle exit, apoptosis, and certain immune functions. Therefore, it is pertinent to explore the role of MLF1 in health and diseases. Studies to date have suggested that MLF1 could act as a double-edged sword, regulating biochemical activities directly or indirectly. In hematopoietic cells, it serves as a protective factor for the development of lineages, and in malignancies, it serves as an oncogenesis factor. The diversity of its functions depends on the binding partners, including tumor inhibitors, scaffolding molecules, mitochondrial membrane proteins, and transcription factors. Emerging evidence indicates that MLF1 influences immune responses as well. This paper reviews the structure, biological function, and research progress on MLF1 in health and diseases to provide new insights for future research.

## Introduction

Myeloid leukemia factor (MLF) is a poorly characterized family of conserved proteins which earliest member, myeloid leukemia factor 1 (MLF1), is associated with hemopoietic lineage commitment and malignancies. MLF1 has so far been shown to be a double-edged sword, acting as either a tumor suppressor or an oncogene, depending on the context of the cell. MLF1 has been initially described in the leukemic fusion protein NPM-MLF1, which is generated by a rare t(3;5)(q25.1;q34) chromosomal translocation in patients with acute myeloid leukemia (AML) (1), and implicated in the development of AML and myelodysplastic syndrome (MDS) (2). Although the role of NPM in the pathogenesis of leukemia has been well studied (3–6), the contribution of MLF1 to normal hematopoiesis and oncogenesis has not been adequately characterized. Several studies have demonstrated that MLF1 can regulate cell cycle exit and differentiation, promote apoptosis, inhibit proliferation in various cell types, enhance immune function, or impair the lymphocyte population. However, its biochemical activity remains largely unclear. Up to now, no systematic overview of MLF1 studies in pathology and physiology has been published. In this review, we summarized current knowledge of MLF1 and provided a valuable reference for future research.

## MLF1 structure and function

**Figure 1:**
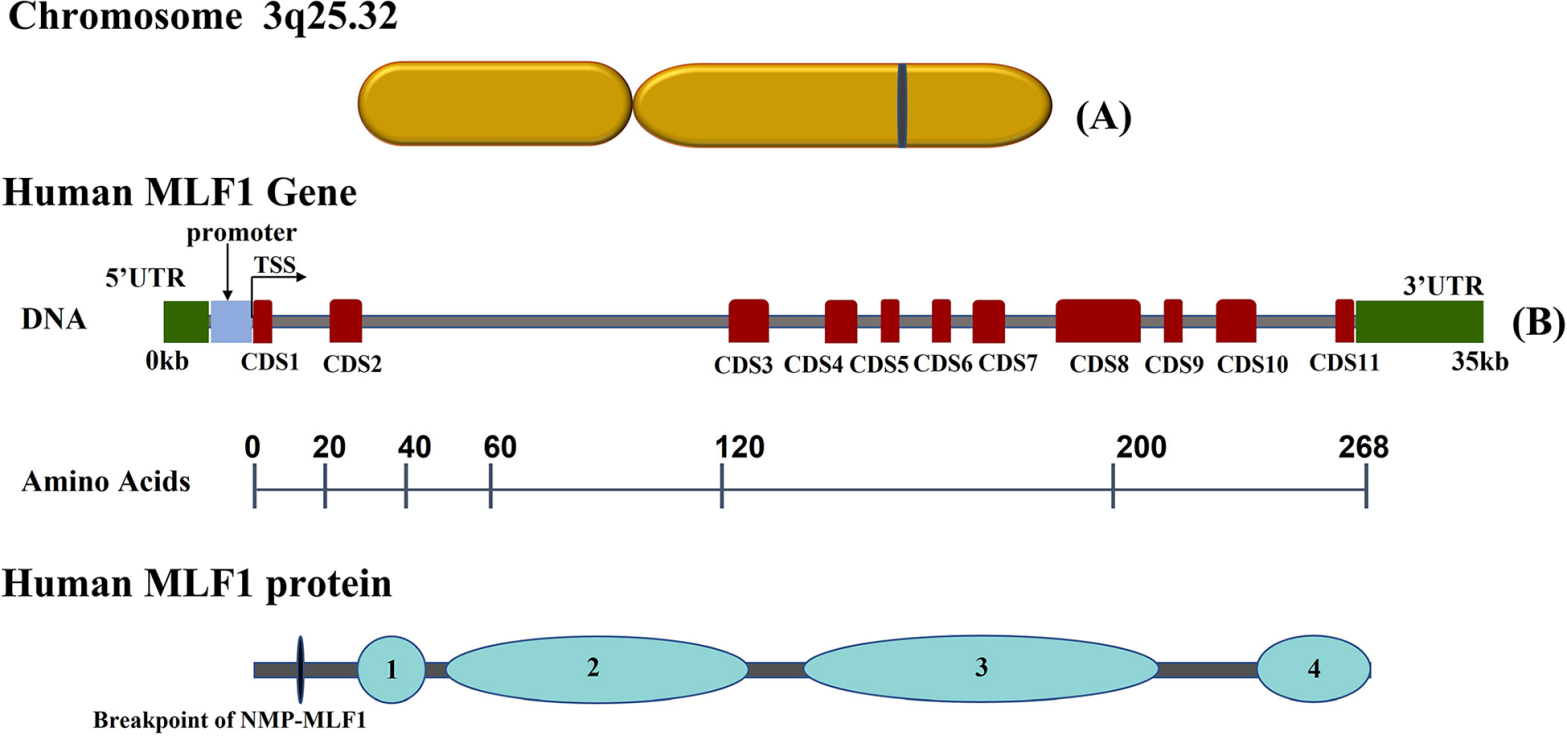
Schematic representation of human Myeloid Leukemia Factor 1 (MLF1) chromosome, gene and corresponding protein domains: **(A)** Diagram of *MLF1* gene chromosome location. **(B)** The grey horizontal line represents the DNA sequence, red boxes on the sequence represent coding sequences (CDS) in MLF1, and two green boxes at the ends indicate UTRs. Underneath, the numbers over the black lines indicate the amino acid positions, which correspond to human MLF1 protein domains ([1] 14-3-3 protein binding domain (Ser34); [2] the COP9 signalosome subunit 3(CNS3) binding domain; [3] MLF family characteristic domain; [4] a SAM domain). All sequences were obtained from the NCBI database.

*MLF1* gene is located on human chromosome 3 and encodes MLF1 protein and its isoforms (7). MLF1 protein is a small nucleocytoplasmic shuttling protein (268 amino acids), which has a functional N-terminal nuclear export signal (NES) and two C-terminal nuclear localization signals (NLS), allowing MLF1 to shuttle between the nucleus and the cytoplasm (8, 9). MLF1 has a characterized central domain preserved within the MLF family (10, 11), comprising two identifiable motifs that bind to 14-3-3 protein and the COP9 signalosome by Ser34 and subunit 3 (CNS3), and a SAM domain, which is involved in many different biological processes and has RNA binding properties (12). Above features of MLF1 are summarized in **Figure 1**. MLF is highly conserved across species from Drosophila, murine, and shrimp to humans (13–15). The phenotypic defects associated with MLF loss in Drosophila can be rescued by human MLF1 (16). MLF overexpression reduces Drosophila wing and eye size (17), which is demonstrated by the fact that MLF activates the bsk-JNK pathway by interacting with DREF (18). Additionally, overexpressed MLF causes abnormal DNA synthesis in Drosophila (19). Enforced expression of murine MLF1 suppresses a rise in the cell cycle inhibitor p27Kip1 to disturb the development and the differentiation of erythrocytes (20, 21). Microarray analysis performed with MLF1-expressing cells has concluded that MLF1, when expressed in the nucleus, inhibited calcium cycle proteins and CR6 (cytokine response protein) associated with differentiation and growth arrest (8). Immune function is also associated with MLF1. It has been identified in kuruma shrimp and characterized as MjMLF, which plays a critical role both in antiviral and antibacterial immunity. MjMLF could inhibit the lethal white spot syndrome virus (WSSV) replication *in vivo* and accelerate *Vibrio anguillarum*, a gram-negative bacteria, clearance in shrimp (22, 23). In contrast, a study about lymphoma has shown that the overexpression of MLF1 increases lymphocyte apoptosis *in vitro* (13). Furthermore, MLF1 absence is consistently associated with the expansion of B- and T-cell numbers in the spleen (24). These findings imply that MLF1 might function as a context-dependent factor involved in the regulation of normal physiological processes and that its absence or overexpression leads to disease.

## MLF1 and its distribution

MLF1 is widely expressed in different tissues. It is highly presented in the testis, heart, lung, brain, thyroid gland, gall bladder, kidney, and digestive system and is expressed to some extent in human bone marrow, spleen, and lymph nodes (25). At the cellular level, MLF1 transcripts are dominantly expressed in CD34+ cells but only slightly in GlyA+, CD3+, CD19+, or CD14+ cells and granulocytes (2). These facts indicate that the expression of MLF1 in CD34+ progenitor cells decreases during differentiation to each lineage, especially toward the myeloid and erythroid lineage (15). Cells at an early stage seem to need MLF1. At the subcellular level, MLF1 is mostly found in the cytoplasm. The apoptosis-inducing domain contained in MLF1 is unique because it requires dimerization and nuclear transportation to induce cell death, whereas most of the well-known ‘death domains’ function in the cytoplasm (26). However, the relationship between the increased accumulation of MLF1 in the cytoplasm and diseases is still unclear. Notably, a functional NES sequence is important for both MLF1 protein and NMP-MLF1 fusion protein to exert proleukemic effects. Additionally, studies have demonstrated that an MLF1 mutant containing only NES sequence inhibited proliferation more strongly than WT protein (9, 27). The contribution of NPM to NPM-MLF1-induced leukemogenesis is debatable (28), whereas NPM-MLF1 fusion protein without NES sequence loses oncogenic transformation ability (9). However, the regulatory mechanisms of the abnormal localization of MLF1 in the nucleus remain unknown.

## MLF1 networks

**Figure 2:**
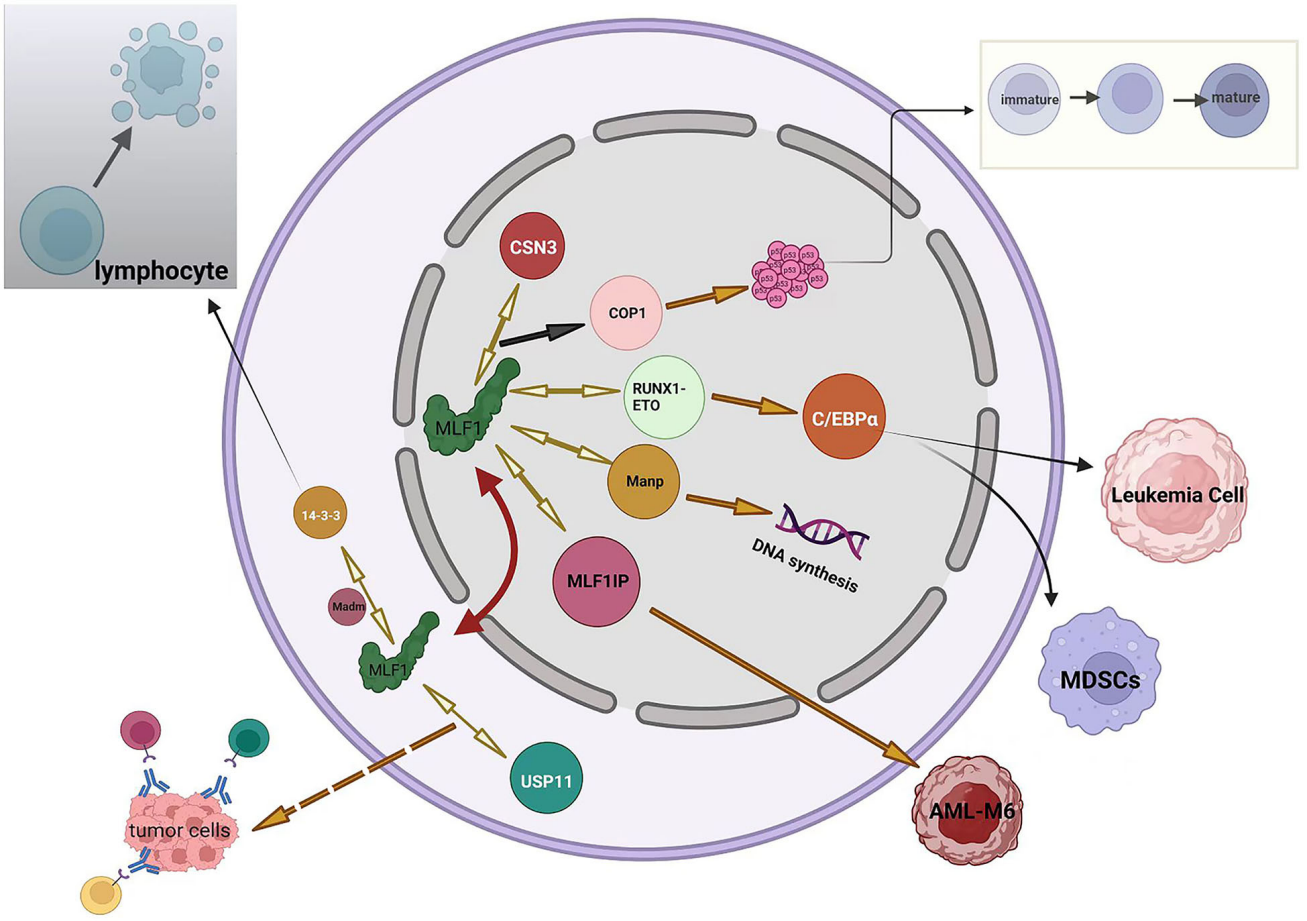
Summary of MLF1 networks. Black arrows represent negative regulation (including inhibition or downregulation), orange arrows represent positive regulation (including promotion or upregulation), orange dotted arrow represents speculation, and bidirectional yellow arrows indicate proteins interacting with MLF1 protein. The red two-way arrow illustrates the shuttling of MLF1 between the nucleus and the cytoplasm.

MLF1 plays an essential role in cell development by interacting with multiple factors, which are summarized in **Figure 2**.

**1. MLF1 in cell development and apoptosis.** MLF1, shuttling from the cytoplasm to the nucleus, binds COP9 subunit 3 (CSN3), which leads to the downregulation of COP1; therefore, the cell cycle of hematopoietic cells becomes arrested because the bonding accelerates the accumulation of wild-type p53 in the nucleus (29). The above-mentioned process has also been demonstrated in Drosophila (17). Tumor suppressor p53 remains a vital mechanism of inhibiting tumor escape from apoptosis, and emerging evidence suggests that mutant p53 also promotes inflammation and supports tumor immune evasion (30, 31). Yoneda-Kato et al. have demonstrated that the MLF1-induced growth arrest depended on the integrity of the p53 allele (29). This raises the question of whether MLF1 still acts as a protective mechanism when p53 is mutated or if it enhances the oncogenicity of mutated p53. Overexpression of MLF1 promotes apoptotic death of the cells but is negatively regulated by 14-3-3 protein blocking its Bcl-XL homology domain 3 (BH3), which prevents the cell from apoptosis (32, 33). 14-3-3 (RSXSXP) motifs are involved in important cell processes, such as death, differentiation, and division (34–36). Bcl-XL, a Bcl-2 family member, maintains a fully functional immune system that ensures an efficient clearance of senescent cells (37). The above-presented conclusion has been obtained in lymphocytes, suggesting that MLF1 is required for lymphocytes to respond to apoptotic stimulations. Additionally, the nuclear content of MLF1 is also regulated by 14-3-3 protein, which sequesters MLF1 in the murine cytoplasm (32), However, another study has suggested the opposite conclusion that the distribution of full-length human MLF1 is 14-3-3 protein-independent (38). Therefore, the subcellular localization of MLF1 is probably regulated by other unknown proteins. A yeast two-hybrid screen has identified that MLF1 binds with an adaptor, which contains a 220-bp cDNA fragment and several potential phosphorylation sites in the vicinity of both the NLS and NES. At the time of its isolation, it had no homology to sequences in the database and was named Madm for MLF1-adaptor molecule. Madm mediates phosphorylation of 14-3-3 binding site of MLF1, which then immunoprecipitates and localizes to the cytoplasm. Thus, Madm might regulate the localization of MLF1 in the cytoplasm. In contrast to MLF1, which promotes the maturation, ectopic expression of Madm suppresses differentiation in myeloid cells (32). Louise N et al. have reported that MLF1 interacts with Manp, also known as scaffold attachment factor-A (SAF-A), which is a member of the heterogeneous nuclear ribonucleoprotein (hnRNP) family and homologous to hnRNP-U. Manp localizes exclusively in the nucleus and redirects MLF1 into the nucleus (8). Recent studies have suggested that hnRNP-U regulates DNA replication, organizes large-scale chromosome structures, and protects the genome from instability (39–41). The effects of MLF on DNA synthesis have been previously discussed (19). However, the relationship between MLF1 and hn-RNP in DNA synthesis remains unclear.

**2. MLF1 in immune function and leukemia.** In a drosophila model of leukemia, MLF has been demonstrated to control the development of hematopoietic stem cells by stabilizing the RUNX transcription factor Lozenge (LZ). MLF controls LZ activity and prevents its degradation, which is critical to control crystal cell number in the fly (42). Further study has shown that MLF and DnaJ-1 interact through conserved domains to form a chaperone complex that directly regulates LZ activity. Importantly, the interaction controls RUNX transcription factor activity and Notch signaling during blood cell development *in vivo* (43). RUNX members are key regulators of hematopoiesis; particularly, RUNX1 functions as a positive regulator for definitive hematopoietic stem cell emergence and megakaryocyte and lymphocyte differentiation (44). RUNX1-ETO, the mutant and infusion form of the RUNX1 protein, has been identified in cancer. MLF1 stabilizes the human oncogenic fusion protein RUNX1-ETO. Further study has indicated that MLF1 impairs RUNX1-ETO accumulation and reduces RUNX1-ETO-dependent leukemia cell proliferation (42). It is reasonable to conclude that MLF1 functions as a tumor suppressor gene in leukemia. However, the expression level of MLF1 in healthy adults’ bone marrow is not as high as expected. Moreover, high expression of MLF1 is associated with poor prognosis for AML and MDS (2). To some extent, MLF1 is required to inhibit the development of leukemia. However, it does not always appear to be a protective factor, and when leukemia is developed, MLF1 is positively correlated with leukemia (2). Reasonably, it can be inferred that *MLF1* is a context-dependent gene, with its elevated expression being associated with leukemia promotion and suppression in different settings. CCAAT/enhancer-binding protein-α (C/EBPα) is a key transcription factor regulating myeloid differentiation in normal hematopoiesis and is frequently dysregulated in AML (45). Studies have shown that Trib1 and RUNX1-ETO downregulate C/EBPa and induce AML in mouse models (46, 47). MLF1 treatment upregulates the level of C/EBPα by suppressing Trib1 or RUNX1-ETO, which causes the inactivation of myeloid-derived suppressor cells (MDSCs) with potent antitumor responses across different tumor models and cancer patients (48). In mouse or leukemia cell models, the distribution of C/EBPa is paralleled with MLF1 (49). MLF1-interacting protein (MLF1IP), also known as PB1P1, KLIP1, KLP1, CENP-U, and CENP-50, specifically binds with MLF1, as shown by yeast two-hybrid analysis and pulldown assays (50). MLF1IP differs from MLF1 without other known protein homology. MLF1IP is a centromere-binding protein (51) that shows 25% identity to the SMC family of proteins and some homology to myosin, which is involved in actin cytoskeletal organization (52). *MLF1IP* may be an erythroid lineage-specific gene, as it is expressed exclusively in CFU-E erythroid precursor cells but not in mature erythrocytes (50, 53). MLF1 drives the occurrence of erythroleukemia as well (20, 21). A study has found that NMP-MLF1 infusion protein is more likely to occur in M6 (according to FAB classification) patients than in other leukemia types (54). Therefore, the interaction between *MLF1IP* and *MLF1* will most likely play a role in the occurrence of M6. High expression of *MLF1IP* is associated with poor prognosis in several cancers, such as breast cancer, glioma, and diffuse large B-cell lymphoma (DLBC) (55). Furthermore, *MLF1IP* also plays a role in the development of the immune system (56). However, the functional consequences of *MLF1* and *MLF1IP* interaction remain largely unknown. HAX-1, a 35-kDa inner mitochondrial membrane protein, functions as an anti-apoptosis protein (57), and its deficiency and overexpression result in the loss of lymphocytes and tumorigenesis, respectively (58, 59). This expression balance of MLF1 is also critical for its function. MLF1 has been recently revealed to directly associate with HAX-1 by co-immunoprecipitation assay. Animal experiments have confirmed that the two have interaction, and severe splenocyte and thymocyte lymphopenia in Hax1−/− mice can be reversed by MLF1 deficiency (13). However, it is unclear whether their effects on lymphocytes are synergistic or antagonistic. As of now, despite conflicting evidence, the relationship between MLF1 and immune function has not been adequately investigated. Further research is required to clarify this issue.

**3. MLF1 in antitumor protection.** MLF1 protein is directly associated with the deubiquitinase ubiquitin-specific peptidase 11 (USP11), which is a promising therapeutic target. Additionally, USP11 has promoted the accumulation of MLF2 in all tested cells (60), whereas MLF1 and MLF2 are approximately 40% similar (11). USP11 plays a dual role in the development of tumors (61, 62). Based on the studies conducted so far, *MLF1* may act as both a tumor suppressor and tumor oncogene, depending on the context of the cell. It is worth mentioning that MLF1 is a positive factor in various biological processes, such as progenitor cell development and tumor regression. Whether MLF1, together with upregulated USP11 protein, enhances antitumor ability still needs further research.

## MLF1 and disease

MLF1 functions as a double-edged sword in various diseases. An early clinical study has found that t(3;5) is more likely to occur in M6 patients than in patients with other leukemia types (54). A preclinical study has confirmed that MLF1 expression drives the occurrence of erythroleukemia (20, 21). A significantly higher level of MLF1 expression is detected in over 25% of patients with immature AML subtypes and higher malignant MDS (2). MLF1 is also upregulated in lung squamous cell carcinoma and esophageal carcinomas (63, 64). MLF1 overexpression results in aggregate formation; however, there is still controversy over the cause and effect of protein aggregate in neurodegenerative diseases (65). The whole-exome sequencing of small intestine neuroendocrine tumors has revealed that *MLF1* is therapeutically relevant (66). The presence of MLF1 protein inhibits apoptosis caused by neurotoxicity induced by Huntingtin (HTT) aggregates (67). An extensive genome-wide association study (GWAS) has indicated that *MLF1* expression is high in neuroblastoma and that silenced MLF1 significantly suppresses tumor proliferation (68). Recent research in the heart has shown that the increased expression of *MLF1* leads to accelerated apoptosis and reduced cardiac cell proliferation (69). However, an aberrant downregulation of *MLF1* is also related to tumorigenesis. Aberrant DNA methylation plays a significant role and is extensive (70), as indicated by the higher incidence of aberrant DNA methylation of known tumor-suppressor genes than that of mutations (71). *MLF1* is methylation-silenced in the gastric cancer cell line and is upregulated 27-fold after 5-AZA-dC treatment. There is a possibility that *MLF1* silencing is causally related to the development and progression of gastric cancer (72, 73). A comparative study has identified that *MLF1* is also a methylation marker for the detection of early gastric neoplasia and field cancerization (74). Shuang Zhao et al. have found that the expression levels of *MLF1* were downregulated in tumor tissues compared to normal tissues, which suggested that *MLF1* influences tumor initiation and progression in nasopharyngeal carcinoma (75). Defects in the centrosome and cilium are associated with a set of human diseases. Ramona A. Hoh et al. have found that MLF1 was associated with diseases, was upregulated during ciliogenesis, and localized to centrioles and cilia (76). Hypermethylated *MLF1* gene in mantle cell lymphoma (MCL) has been confirmed by genome-wide DNA methylation analysis, and aberrant methylation is associated with inverse changes in mRNA levels (77). Marcela B. Mansur et al. have identified a recurrent somatic deletion on chromosome 3. This loss results in the complete deletion of MLF1 and has not been previously described in infant T-cell acute lymphoblastic leukemia (78).

## Conclusion and future perspectives

In conclusion, MLF1 is a small shuttling protein playing a critical role in biological and pathological processes. Currently, research regarding MLF1 has mainly focused on cancer development, which is still an obscure and disputed topic. In general, although there is more evidence supporting the point that MLF1 contributes to tumor suppression, a few studies have confirmed the tumorigenesis of MLF1 in solid and hematologic tumors, which cannot be neglected. Additionally, the functions of MLF1 in immune response still need further investigations despite some already reported studies. Given the complexity and variety of involved proteins, we may draw a conclusion that MLF1 might be a double-edged factor in the regulation of cell cycle, immunity, stem cell development, and cancer. However, studies about MLF1 are still inadequate; therefore, expanding the research on MLF1 is significant and may enrich the knowledge of MLF1 in the above-mentioned conditions. On the other hand, exploring regulations of MLF1 shuttling will provide a better understanding of MLF1, which helps develop novel specific MLF1-aimed drugs that might provide a promising strategy for cancer treatment, as well as other pathologies, such as neurological diseases. Therefore, analysis of the partner protein, localization, and shuttling mechanism might provide new insights for future research.

## Author contributions

All authors contributed to the article and approved the submitted version.
